# Supplementary material for: Highly Expressed Progesterone Receptor B Isoform Increases Platinum Sensitivity and Survival of Ovarian High-Grade Serous Carcinoma
Source: Cancers (Basel). 2021 Nov 8;13(21):5578. doi: 10.3390/cancers13215578 (PMC8582698; doi:10.3390/cancers13215578)
Supplement: Supplementary file 1 [file cancers-13-05578-s001.zip › cancers-1421508-supplementary.pdf]

cancers MDPI

Supplementary Materials

Highly Expressed Progesterone Receptor B Isoform Increases Platinum Sensitivity and Survival of Ovarian High-Grade Serous Carcinoma

Hao Lin, Kuo-Chung Lan, Yu-Che Ou, Chen-Hsuan Wu, Hong-Yo Kang, I-Chieh Chuang and Hung-Chun Fu

Table S1. Univariate and multivariate Cox regression analysis of the risk factors for progression-free survival.

	**Univariate Analysis**			Multivariate Analysis		
Variable	HR	95% C.I.	*p* value	HR	95% C.I.	*p* value
Age ≥ 60	1.18	0.71 – 1.97	0.529			
Menopause	1.14	0.69 – 1.89	0.611			
Stage III, IV	3.17	1.37 – 7.36	0.007	1.99	0.82 – 4.83	0.129
Sub-optimal debulking	2.73	1.65 – 4.49	<0.001	2.2	1.31 – 3.70	0.003
CA125 ≥ 500 U/mL	1.72	1.05 – 2.82	0.032	1.35	0.81 – 2.26	0.244
PR-B score < 12.5	1.95	1.09 – 3.49	0.024	1.79	0.99 – 3.22	0.052

Abbreviations: CA-125: carbohydrate antigen-125, C.I.: confidence interval, HR: hazard ratio, PR-B: progesterone receptor-B.

Table S2. Univariate and multivariate Cox regression analysis of the risk factors for cancer-specific survival.

	**Univariate Analysis**			**Multivariate Analysis**		
Variable	HR	95% C.I.	*p* value	HR	95% C.I.	*p* value
Age ≥ 60	1.94	1.06 – 3.57	0.032	2.12	0.96 – 4.69	0.061
Menopause	1.31	0.69 – 2.49	0.407			
Stage III, IV	1.71	0.72 – 4.06	0.222			
Sub-optimal debulking	2.71	1.46 – 5.03	0.002	2.45	1.26 – 4.77	0.008
CA125 ≥ 500 U/mL	1.82	0.98 – 3.38	0.059			
PR-B score < 12.5	2.15	0.99 – 4.65	0.051			

Abbreviations: CA-125: carbohydrate antigen-125, C.I.: confidence interval, HR: hazard ratio, PR-B: progesterone receptor-B.

Copyright: © 2021 by the authors. Licensee MDPI, Basel, Switzerland. This article is an open access article distributed under the terms and conditions of the Creative Commons Attribution (CC BY) license (http://creativecommons.org/licenses/by/4.0/).
